# An Intact Kidney Slice Model to Investigate Vasa Recta Properties and Function in situ

**DOI:** 10.1159/000339110

**Published:** 2012-07-20

**Authors:** C. Crawford, T. Kennedy-Lydon, C. Sprott, T. Desai, L. Sawbridge, J. Munday, R.J. Unwin, S.S.P. Wildman, C.M. Peppiatt-Wildman

**Affiliations:** ^a^Medway School of Pharmacy, The Universities of Kent and Greenwich at Medway, Chatham, London, UK; ^b^Urinary System Physiology Unit, Royal Veterinary College, London, UK; ^c^UCL Centre for Nephrology, Royal Free Hospital, University College London, London, UK

**Keywords:** Kidney, Microvasculature, Medulla, Hemodynamics, Tubulovascular crosstalk, Innervation, Tissue slicing, Live kidney slice model

## Abstract

**Background:**

Medullary blood flow is via vasa recta capillaries, which possess contractile pericytes. In vitro studies using isolated descending vasa recta show that pericytes can constrict/dilate descending vasa recta when vasoactive substances are present. We describe a live kidney slice model in which pericyte-mediated vasa recta constriction/dilation can be visualized in situ.

**Methods:**

Confocal microscopy was used to image calcein, propidium iodide and Hoechst labelling in ‘live’ kidney slices, to determine tubular and vascular cell viability and morphology. DIC video-imaging of live kidney slices was employed to investigate pericyte-mediated real-time changes in vasa recta diameter.

**Results:**

Pericytes were identified on vasa recta and their morphology and density were characterized in the medulla. Pericyte-mediated changes in vasa recta diameter (10–30%) were evoked in response to bath application of vasoactive agents (norepinephrine, endothelin-1, angiotensin-II and prostaglandin E_2_) or by manipulating endogenous vasoactive signalling pathways (using tyramine, *L*-NAME, a cyclo-oxygenase (COX-1) inhibitor indomethacin, and ATP release).

**Conclusions:**

The live kidney slice model is a valid complementary technique for investigating vasa recta function in situ and the role of pericytes as regulators of vasa recta diameter. This technique may also be useful in exploring the role of tubulovascular crosstalk in regulation of medullary blood flow.

## Introduction

Regulation of renal medullary blood flow in physiological and pathophysiological situations is still poorly understood because of the relative inaccessibility of the medullary microcirculation in vivo. In vitro studies using isolated descending vasa recta (DVR) have proposed that several local and systemic factors, such as endothelins (ET), eicosanoids, nitric oxide (NO), angiotensin-II (Ang-II), adenosine, and ATP, may be involved in its regulation [1]. Regional perfusion studies in which laser Doppler optical fibres have been placed in the renal parenchyma to measure autoregulation of medullary blood flow suggest a link between changes in blood flow, extracellular fluid volume regulation, and salt and water excretion [2,3,4,5]. It is also thought that the medullary circulation (vasa recta capillaries) is autoregulated (in DVR capillaries over the range of 85–160 mm Hg) and that changes in medullary blood flow are not simply a passive response to changes in renal artery blood flow or pressure; although exactly how this is achieved is still unclear [6]. Furthermore, this subject is not without controversy, the concept of medullary autoregulation has yet to be widely accepted by the field.

Until recently, regulation of tissue blood flow was thought to occur at the level of the arteries and pre-capillary arterioles; however, it has been shown that specialized contractile smooth muscle-like pericyte cells found on capillaries can control capillary diameter in situ in whole retina and in cerebellar brain slices [7]. This is also relevant to the renal medulla, because pericyte cells are present along DVR capillaries [8]. In vitro studies using isolated DVR suggest that the medullary microcirculation is likely to be regulated by locally released vasoactive agents independent of any changes in cortical blood flow [9,10,11,12,13,14]. Moreover, changes in medullary blood flow are thought to play an important part in the pathogenesis of acute kidney injury [15], and medullary tubulovascular crosstalk could be critical to the control of medullary blood flow in health and disease.

Since the medulla is not readily accessible to direct observation in vivo, except in neonatal rats where the papilla can be exposed by opening the renal pelvis [16], there have been no studies of DVR regulation in the intact medulla, although it is known that pericytes respond to exogenous application of vasoactive agents that can alter isolated DVR diameter [17]. The inaccessibility of the intact medulla means that key endogenous regulators of DVR blood flow have not been investigated in the intact kidney in situ or in vivo. To address this issue, we have developed a ‘live’ intact kidney slice model to investigate pericyte-mediated regulation of vasa recta diameter in situ in the renal medulla. A potential advantage of the kidney slice model is that although vasa recta are not perfused, the surrounding tubules and local innervation remain intact, and their contributing effects on vasa recta diameter can be explored.

## Materials and Methods

### Tissue Slicing

Animal experiments were conducted in accordance with United Kingdom Home Office Scientific Procedures Act (1986). Adult male Sprague-Dawley rats (250–300 g, purchased from Charles River UK Ltd, Kent, UK) were killed by cervical dislocation; their kidneys were immediately removed and placed in ice-cold physiological saline solution (PSS) bubbled with 95% O_2_/5% CO_2_ and prepared for slicing. Prior to slicing, the kidneys were de-capsulated and any renal artery remnants removed. A single kidney was secured on the slicing block of a vibratome tissue slicer (Leica model VT1200S; Leica Microsystems (UK) Ltd, Milton Keynes, Bucks., UK), and submerged in a bath of ice-cold PSS bubbled with 95% O_2_/5% CO_2_. PSS contained (m*M*) 100 NaCl, 5 KCl, 0.24 NaH_2_PO_4_, 0.96 Na_2_HPO_4_, 10 Na acetate, 1 CaCl_2_, 1.2 MgSO_4_, 5 glucose, 25 NaHCO_3_, 5 Na pyruvate (Sigma-Aldrich Ltd, Poole, Dorset, UK). The pH was adjusted to 7.4 using 10 *M* NaOH. The outer cortical dome region (∼3 mm tissue) of the kidney was removed to expose the top of the renal medulla and serial 200 µm-thick coronal kidney slices (in which there was intact cortex and medulla) were cut. Slices were collected and maintained at room temperature in a holding chamber containing PSS, and bubbled with 95% O_2_/5% CO_2_ to conserve tissue viability. The slices to be used in ‘live’ experiments were maintained for up to 3 h in the holding chamber, or slices were fixed with 4% paraformaldehyde (Sigma-Aldrich Ltd) and used for immunohistochemistry.

### Tissue Viability

To confirm the viability of kidney slices, an on-stage perfusion system was used to load fluorescent dyes into the slices to label all cells (Hoechst 33342) and dead cells (propidium iodide (PI)). Non-fixed kidney slices were secured with a slice anchor in an open-bath chamber (∼1.5 ml volume) (Harvard Apparatus, Edenbridge, Kent, UK) and superfused with PSS bubbled with 95% O_2_/5% CO_2_ at 5 ml/min. Slices were imaged using a Zeiss 510 NLO Axiovert microscope coupled to a tuneable Coherent Chameleon laser. Prior to imaging of kidney slices, we established the optimum excitation wavelength for each relevant probe imaged using the 2-photon laser, by generating a 2-photon excitation spectrum. Hoechst 33342 (Invitrogen Ltd, Paisley, UK) was used at 5 µ*M* to label all cell nuclei and was excited at 720 nm. PI (Sigma-Aldrich Ltd) is taken up by cells with compromised cell membranes and was used at 20 µ*M* to label the nuclei of all dead cells and was excited at 543 nm with a single photon laser. Emitted light was collected with the following filters: band-pass 435–485 nm (Hoechst 33342) or long-pass 560 nm (PI). Image processing was performed using Zeiss LSM image browser software (Carl Zeiss Ltd, Welwyn Garden City, Herts., UK). The ratio of live:dead cells (Hoechst labelled:Hoechst-PI co-labelled, respectively) was also calculated using Zeiss LSM image browser software.

To further validate cell viability, and to distinguish between tubular and vascular cells, live kidney slices were incubated for 20 min with the cell-permeant calcein-AM dye. This non-fluorescent dye is converted to a bright green fluorescence by intracellular esterases following uptake into live cells [18]. Calcein-AM (Invitrogen Ltd) was used at 2 µ*M* (in 0.002% pluronic acid) as a vital stain and excited at 800 nm; emitted light was collected using band-pass 500–550 nm. Image processing was performed using Zeiss LSM image browser software, as before.

### Immunohistochemistry

Live kidney slices were incubated with Alexa Fluor 488-conjugated isolectin B_4_ (IB_4_) (Invitrogen Ltd) – a marker for α-*D*-galactosyl residues – to identify the vasa recta capillaries. IB_4_ (50 µg/ml) was prepared in PSS bubbled with 95% O_2_/5% CO_2_; slices were incubated with IB_4_ for 45 min, washed with PSS and subsequently fixed using 4% paraformaldehyde. Fixed kidney slices were then incubated for 16 h with the anti-neural-glial 2 (NG2) polyclonal antibody (Millipore UK Ltd, Watford, UK). The anti-NG2 antibody, previously used to identify pericytes in the CNS [7], was probed with an Alexa Fluor 555-conjugated donkey anti-rabbit secondary antibody (Invitrogen Ltd). For experiments in which pericytes were co-localized with sympathetic nerves, slices were incubated with anti-NG2 and anti-tyrosine hydroxylase (Vector Laboratories Ltd, Peterborough, UK) primary antibodies, respectively. The tyrosine-hydroxylase signal was amplified with a biotinylated secondary antibody and probed with a FITC-conjugated tertiary antibody (Fluorescent Avidin Kit; Vector Laboratories Ltd). Kidney slices were mounted using Citiflour (Agar Scientific Ltd, Stanstead, Essex, UK) and the medulla of fixed slices were imaged using a Zeiss LSM 510 laser scanning confocal microscope (Carl Zeiss Ltd). Alexa 488-conjugated IB_4_ and FITC-conjugated secondary antibodies were excited at 488 nm and Alexa Fluor 555-conjugated secondary antibody excited at 543 nm. Emitted light was collected with the following filters: long-pass 560 nm (Alexa Fluor 555) and band-pass 505–550 nm (FITC and IB_4_). Pericytes on the vasa recta were imaged in both the inner and outer medulla.

Confocal images of fluorescently labelled pericytes (Alexa 555, red) and vasa recta capillaries (Alexa 488, green) were used to: (i) calculate the density of pericytes in both the inner and outer medulla, (ii) to measure the average primary 1° process length [19] and cell body length of pericytes in both regions. For density calculations, ≥3 × 100-µm^2^ regions in both the inner and outer medulla of every kidney slice were selected at random (n = 20 for inner and outer medulla). Measurements of pericyte cell body length and 1° process lengths in both the outer and inner medulla were measured using Zeiss LSM image browser software.

### Functional Experiments and Analysis

Live kidney slices were secured in an open-bath chamber using a purpose-built platinum slice anchor and transferred to the stage of an upright Olympus microscope (model BX51WI; Olympus Microscopy, Southend-on-Sea, Essex, UK). Kidney slices were continuously superfused (∼2.5 ml/min, 1.25 ml bath volume) with PSS, bubbled with 95% O_2_/5% CO_2_ and maintained at room temperature. Pericytes on the vasa recta capillaries (vasa recta were defined as <10 µm in diameter) were identified by their previously established ‘bump-on-a-log’ morphology [7], and DIC images were captured through an Olympus 60× water immersion objective (0.9 NA). Real-time video images of changes in vasa recta diameter were collected every second by an attached Rolera XR CCD camera and recorded using Image Pro Software (Media Cybernetics Inc., Marlow, Bucks., UK). Live kidney slices were superfused with the following vasoactive compounds: Ang-II (10 and 100 n*M*; see online supplementary video 1, www.karger.com?doi=10.1159/000339110), norepinephrine (NE; 10 n*M*), endothelin-1 (ET-1; 10 n*M*), indomethacin (30 µ*M*), and *L*-N^G^-nitroarginine methyl ester (*L*-NAME; 1 m*M*), to evoke vasoconstriction, and the NO donor, S-nitroso-N-acetyl-*D, L*-penicillamine (SNAP; 100 µ*M*) and prostaglandin E_2_ (PGE_2_; 10 µ*M*) were used to evoke vasodilation. All vasoactive compounds were purchased from Sigma-Aldrich Ltd), and working solutions were prepared in oxygenated PSS. In experiments in which ATP release (from tubules) was stimulated, a hypotonic PSS solution (NaCl concentration reduced by 5%) was applied to live kidney slices.

Time-series analysis of live kidney slice experiments was carried out using the public domain software ImageJ (NIH, http://rsb.info.nih.gov.ij). For each experiment, both a pericyte site and a non-pericyte site were identified on a single vasa recta. The diameter of the vasa recta at both locations was measured every 5 frames for the duration of the experiment (1 frame = 1 s). Typically, kidney slices were superfused with PSS alone for 70 s to establish a baseline vessel diameter at the pericyte and non-pericyte site. Slices were then exposed to an agonist/antagonist to evoke a change in vasa recta diameter, and were then subjected to a PSS wash (see online suppl. video 1). Each capillary acted as its own control; an average of the first 5 measurements was taken to represent the resting baseline diameter value (D_b_), and expressed as 100%, for both pericyte and non-pericyte sites. All subsequent diameter measurements (D) were calculated and expressed as a percentage of the corresponding baseline value for both pericyte and non-pericyte sites (see equations 1 and 2). Percentage change in vessel diameter was calculated from actual vessel diameter measurements made throughout each experiment; the smaller changes in a vessel diameter reported here (e.g. 2% of 9.5 µm vessel) are calculated rather than measured changes. In our experiments, we calculate that 10 µm is defined by 47 pixels, which is measurable.

(1) $$\%\delta \text{Vessel diameter}=\left(\frac{\text{measured diamete}\left(\text{D}\right)}{\text{mean baseline diameter}{\left(\text{D}\right)}_{\text{b}}}\right)\times 100\left(1\right)$$

(2) %constriction or dilation=100%(baseline)-%δvessel diameter(2)

For all experiments, statistical significance was calculated using Student's t test; p < 0.05 was considered significant. Values are expressed as mean ± SE. N numbers displayed represent number of pericytes (1 pericyte and non-pericyte site per kidney slice), since the variation observed, and found in similar kidney [20] and CNS studies [7], occurred between pericytes and not animals. All experiments were performed in at least 3 different animals. One vasa recta per kidney slice was used to ensure all vessels were ‘naive’ prior to exposure to any drug.

## Results

### Viability of Kidney Tissue Slices

**Fig. 1:**
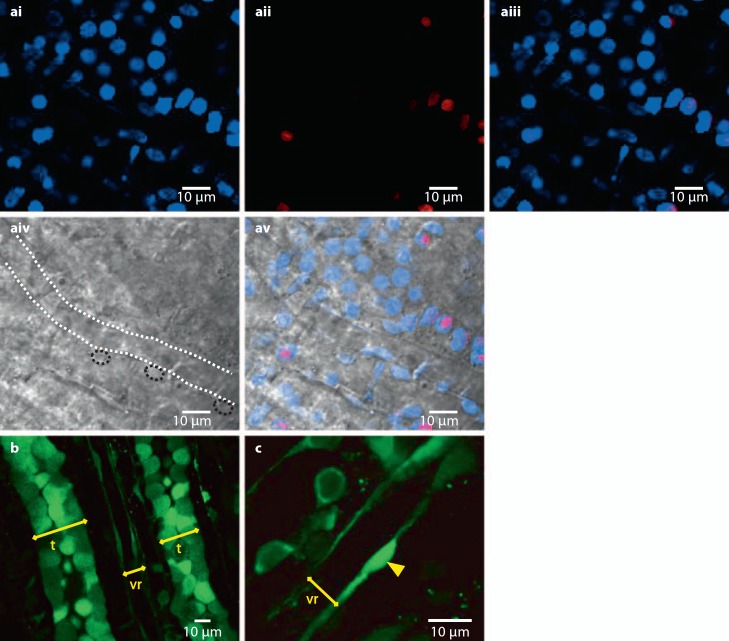
Viability of kidney slices. Confocal images of kidney slices incubated with Hoechst and PI to label nuclei of all (**ai**) and dead (**aii**) cells, respectively. Overlay of images **ai** and **aii** demonstrates the ratio of live:dead cells (**aiii**). The corresponding brightfield image indicates the location of pericytes (**aiv**, black dotted lines) on a single vasa recta capillary (**aiv**, white dotted lines). Overlay of images **ai–aiv** shows all pericytes in this field of view are live (**av**). Calcein uptake is shown in tubules (t) and vasa recta capillaries (vr) in the renal medulla of live kidney slices (**b**). Pericytes (arrowhead) were also labelled by calcein (**c**).

A selection of fluophores was used to assess the viability of the live kidney tissue slices. Figure 1a shows a typical live kidney slice loaded with Hoechst (fig. 1ai) and PI (fig. 1aii), an overlay of Hoechst and PI (fig. 1aiii), a brightfield DIC image of the corresponding area (fig. 1aiv), and an overlay of all images (fig. 1av). In three independent experiments we determined the ratio of Hoechst:Hoechst and PI (live:dead) labelled nuclei to be 5:1 (per 100 µm^2^, n = 5 slices, n = 3 animals), indicating that the majority of tubular and vascular cells within the kidney slice were live, thus confirming the tissue slices were viable (fig. 1aiii). Tubules and vasa recta capillaries loaded with the viable marker calcein and were clearly fluorescent, and therefore considered viable (n = 9 slices, n = 3 animals; fig. 1b). In addition, it was observed that capillaries and vessels consistently run in parallel in the renal medulla. Tubules of different sizes were observed, ranging from 10 to 40 µm in diameter (n = 9 slices, n = 3 animals). The average diameter of vasa recta capillaries was 7.9 ± 0.6 µm (n = 9 slices, n = 3 animals). Pericytes were identified on the outside of capillaries by their previously defined ‘bump-on-a-log’ morphology [7] and they were also fluorescent, indicating that pericytes in our live kidney slices were also viable (fig. 1c, arrowhead).

### Characteristics of Vasa Recta Pericytes in situ

**Fig. 2:**
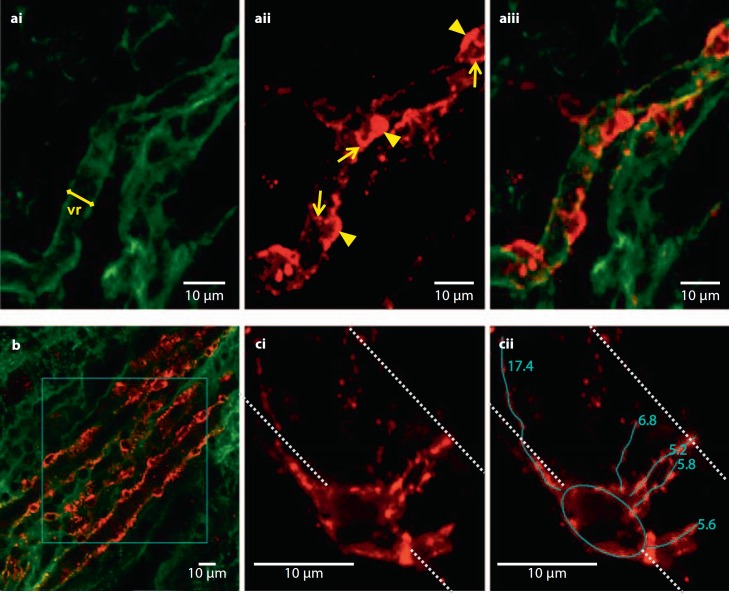
Identification and characterization of pericytes on vasa recta capillaries in the renal medulla. Vasa recta capillaries (vr) were labelled with Alexa-488-conjugated IB_4_ (**ai**, green). The cell body (arrowheads) and processes (arrows) of pericytes in the renal medulla were labelled with anti-NG2 and Alexa-555-conjugated secondary antibody (**aii**, red). An overlay of images **ai** and **aii** identifies pericytes (red) on vasa recta capillaries (green) (**aiii**). The density of pericytes was calculated for both inner and outer medulla, by selecting 100-µm^2^ regions in confocal images of NG2 and IB_4_-labelled kidney slices (**b**). A magnified image of a pericyte (**ci**) shows the cell body and processes (dotted lines indicate outer vessel wall). Cell body length and 1° processes (running along and around the vessel) were measured (**cii**, vessel outline indicated by dotted lines).

Vasa recta and pericytes were labelled with fluorescently conjugated IB_4_ (fig. 2ai) and with anti-NG2 (fig. 2aii), respectively. Confocal images of labelled kidney slices were used to identify the prominent pericytes cell body (bump-on-a-log) and processes that run along and wrap around the abluminal surface of vasa recta capillaries (fig. 2aiii). Pericytes were observed at regular intervals along the vasa recta in both the inner and outer medulla. 1° processes were seen to extend from the pericyte cell body along and around the vasa recta (fig. 2aiii), as previously described in electron microscopy studies [19]. IB_4_ labelling did not distinguish between descending (DVR) and ascending (AVR) limbs.

Wide-field images of vasa recta and pericytes were collected from 3 animals, 3 tissue slices per animal, and pericyte characteristics were determined by analysing 100 µm^2^ areas in both inner and outer medulla regions per kidney slice (fig 2b). The pericyte density per 100 µm^2^ in the outer medulla was significantly greater than that in the inner medulla (n = 20, 100 µm^2^ regions analysed for each region, p < 0.05; table 1). There was no significant difference in pericyte cell body or process length between the inner and outer medulla; the mean pericyte cell body length was 9 µm (n = 99) and mean pericyte process length was 9 µm (n = 158; table 1). The mean distance between pericyte cell bodies was 16 µm (n = 91). Interestingly, process length ranged from 2 to 18 µm, with 1° processes running along the vasa recta and wrapping around the vessel (fig. 2ci, ii).

**Fig. 3:**
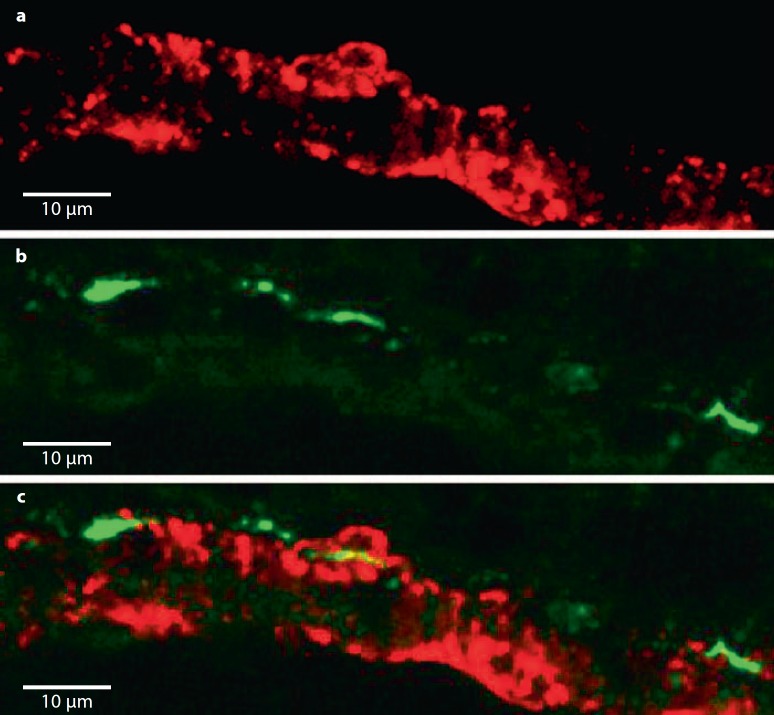
Co-localization of pericytes and sympathetic nerves in the renal medulla. Confocal image of vasa recta pericytes labelled with anti-NG2 and Alexa-555-conjugated secondary antibody (**a**, red). Sympathetic nerve varicosities were identified with FITC-labelled anti-tyrosine hydroxylase (**b**, green). An overlay of images **a** and **b** shows pericytes (red) co-localized with sympathetic nerves (green) on a vasa recta capillary (**c**).

Sympathetic nerve varicosities in the medulla were labelled with an anti-tyrosine hydroxylase antibody and were found to run along vasa recta in close proximity to both inner and outer medullary pericytes (fig. 3). Staining for sympathetic nerves was predominantly detected in the outer medulla, in keeping with the higher density of pericytes found in this area of the medulla.

### Effect of Vasoactive Agents on Vasa Recta Diameter in situ

**Fig. 4:**
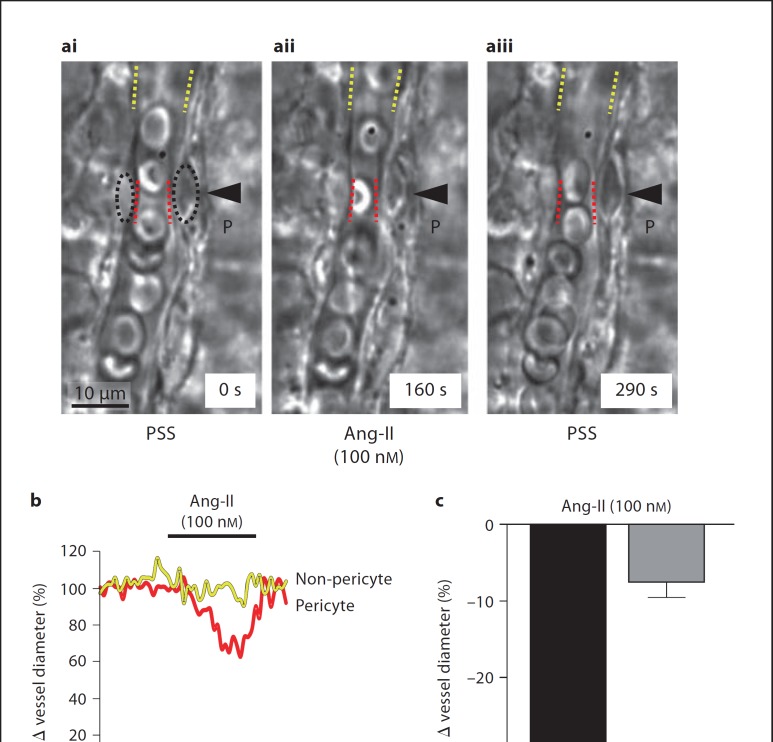
DIC imaging of pericyte-mediated constriction of in situ vasa recta capillaries. Images taken from a time series experiment in which kidney slices were exposed to Ang-II. A typical field of view of a vasa recta capillary superfused with control PSS solution is shown (**ai**). Red blood cells are seen in the lumen and pericytes located on capillary walls (**ai**, arrowhead). Red and yellow dotted lines highlight the regions along the vasa recta where the vessel diameter was measured at pericyte sites and non-pericyte sites, respectively (**ai–iii**). Application of Ang-II (100 n*M*) caused a reduction in vasa recta diameter at the pericyte site (**aii**). Following removal of Ang-II from the perfusate, the vasa recta returned to resting diameter (**aiii**). A representative trace (**b**) of % change in vasa recta diameter at a pericyte site (red trace), and non-pericyte site (grey trace), in response to Ang-II (100 n*M*) exposure. Mean data for these experiments shows Ang-II (100 n*M*) evoked a significantly greater vasoconstriction at pericyte sites (black bar) compared with non-pericyte sites (grey bar); * p < 0.05 (**c**).

**Fig. 5:**
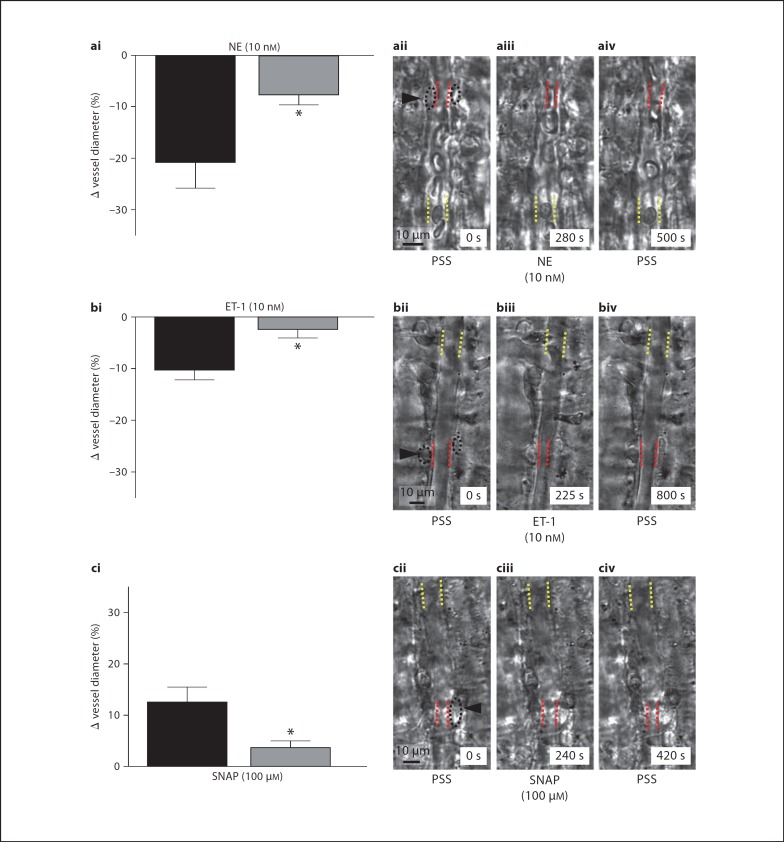
Effect of vasoactive compounds on vasa recta diameter. Superfusion of live kidney slices with NE (10 n*M*), and ET-1 (10 n*M*) evoked a significantly greater constriction of vasa recta at pericyte sites (black bars) than non-pericyte sites (grey bars), **ai** and **bi**, respectively. The NO donor, SNAP (100 µ*M*) evoked a significantly greater dilation of vasa recta at pericyte sites compared with non-pericyte sites (**ci**). Values are mean ± SE, * p < 0.05. Representative images of a pericyte site and non-pericyte site pre-drug exposure (**i**), during superfusion of the drug (**ii**), and during washout of the drug (**iii**), are shown for experiments **a–c**. Pericytes are denoted by black dotted circles, red dotted lines indicate where maximal constriction/dilation occurs at pericyte sites, and yellow dotted lines indicate the corresponding non-pericyte region of the same vasa recta.

The initial resting internal diameter of vasa recta capillaries was 7.5 ± 0.4 µm at pericyte sites, significantly narrower than at non-pericyte sites (9.5 ± 0.4 µm, n >30, p < 0.001). Several vasoactive compounds were tested for their ability to alter vasa recta diameter at pericyte and non-pericyte sites. Changes in vessel diameter were determined by measuring the diameter of the capillary at pericyte and non-pericyte sites before, during, and after the application of a vasoactive compound (see Methods, and figure 4ai–iii and online suppl. video 1 for example of Ang-II-evoked constriction). A representative trace showing the Ang-II (100 n*M*)-evoked change in vessel diameter at a pericyte and corresponding non-pericyte site is displayed in figure 4b. We calculated that, on average, 51% of pericytes responded to agonist exposure by changing vessel diameter. The success rate ranged from 35 to 85% depending on the agonist vessels were exposed to. Ang-II (100 n*M*) caused a significantly greater vasoconstriction (∼4-fold) at pericyte sites (29.3 ± 4.7%) than at non-pericyte sites (7.6 ± 1.9%, n = 14 slices, n = 6 animals, p < 0.001; fig. 4c, online suppl. video 1). Similarly, NE (10 n*M*) significantly decreased vasa recta diameter by 20.8 ± 5.1% at pericyte sites compared with 7.6 ± 2.0% at non-pericyte sites (p < 0.05, n = 7 slices, n = 3 animals; fig. 5ai); ET-1 (10 n*M*) also caused significant vasoconstriction at pericyte sites (10.3 ± 1.9%) compared with non-pericyte sites (2.4 ± 1.7%, n = 6 slices, n = 3 animals, p < 0.05; fig. 5bi). Using Poiseuille's law, which assumes laminar flow, as an approximation [21], and not allowing for the presence of red blood cells [22], the constriction evoked by NE (21%), Ang-II (29%) and ET-1 (10%) would increase resistance to blood flow by 2.7-, 4- and 1.5-fold, respectively. The application of the NO donor SNAP (100 µ*M*) caused a modest but significantly greater dilation of vasa recta at pericyte sites (12.6 ± 2.9%) compared with non-pericyte sites (3.7 ± 1.3%, n = 15 slices, n = 8 animals, p < 0.01; fig. 5ci). Representative images of NE, ET-1 and SNAP-evoked changes in vessel diameter at pericytes are shown in figure 5aii–iv, bii–iv and cii–iv, respectively.

**Fig. 6:**
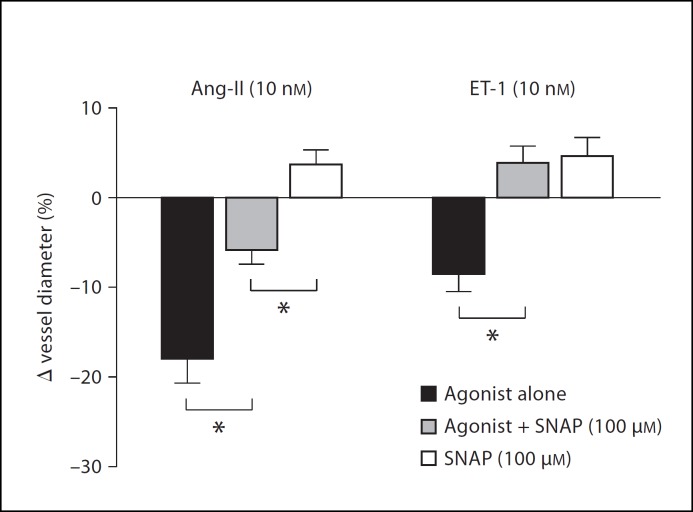
Co-application of SNAP with agonist (Ang-II or ET-1) attenuated the agonist-evoked constriction of vasa recta at pericyte sites. Co-application of SNAP (100 n*M*) with either Ang-II (10 n*M*) or ET-1 (10 n*M*) significantly decreased both the Ang-II- and ET-1-evoked constriction at pericyte sites, by 70% (n = 4) and 150% (n = 10), respectively. Removal of Ang-II (10 n*M*) from the perfusate caused a further dilation of vasa recta at pericyte sites beyond resting diameter, removal of ET-1 (10 n*M*) did not result in any greater dilation. No significant change in the vessel diameter was measured at non-pericyte sites throughout the experiment. Values are mean ± SE. * p < 0.05.

In further experiments, the Ang-II (10 n*M*)-evoked and ET-1 (10 n*M*)-evoked constrictions of vasa recta at pericyte sites was attenuated by the NO donor SNAP (100 µ*M*; fig. 6). Ang-II (10 n*M*) and ET-1 (10 n*M*) evoked an 18.0 ± 2.7% (n = 4 slices, n = 3 animals) and 8.5 ± 1.9% (n = 10 slices, n = 3 animals) constriction at pericyte sites, respectively, which was significantly greater than that measured at non-pericyte sites (2.8 ± 0.7%, p < 0.01, n = 4 slices, n = 3 animals, and 4.7 ± 1.4%, p < 0.05, n = 10 slices, n = 3 animals, respectively). SNAP (100 µ*M*) reduced the Ang-II (10 n*M*)-evoked constriction by 67.7% (to 5.8 ± 1.6%) at pericyte sites (fig. 6), and the ET-1 (10 n*M*)-evoked constriction by 145.9% thus causing vasa recta to dilate to +3.9 ± 1.9% of the original resting diameter at pericyte sites. Removal of Ang-II (10 n*M*) from the perfusate caused a further dilation by SNAP to +3.7 ± 1.6% of the original resting diameter at pericyte sites (p < 0.05, n = 4 slices, n = 3 animals; fig. 6), whereas removal of ET-1 from the perfusate, leaving SNAP alone, failed to evoke a further significant dilation at either pericyte or non-pericyte sites. SNAP also failed to attenuate the Ang-II-evoked constriction of vasa recta at pericyte sites when higher concentrations of Ang-II (100 n*M*) were used (data not shown). No significant changes in vasa recta diameter were observed at non-pericyte sites during these experiments (data not shown).

**Fig. 7:**
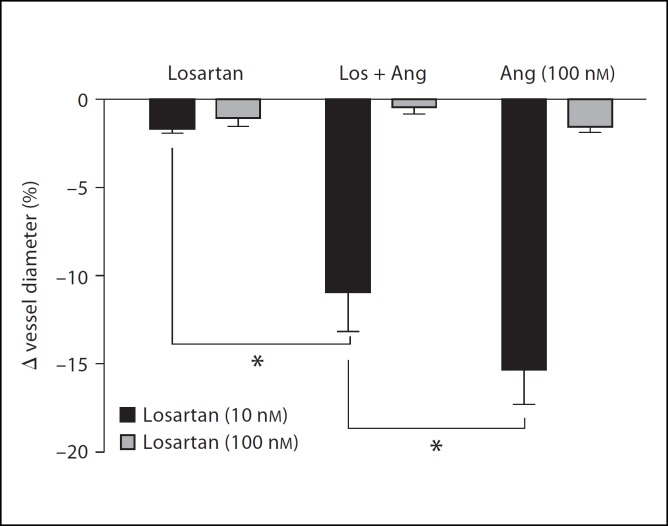
The AT_1_ receptor antagonist, losartan, concentration-dependently blocked the Ang-II (100 n*M*)-evoked constriction of vasa recta at pericyte sites. Losartan alone (10 n*M* or 100 n*M*) had no effect on capillary diameter (n = 8 and n = 9, respectively). Losartan (10 n*M*, black bars) significantly and reversibly attenuated (∼30%) the maximum Ang-II (100 n*M*)-evoked vasoconstriction of vasa recta by pericytes (n = 8). Losartan (100 n*M*, grey bars) significantly and irreversibly blocked vasoconstriction of vasa recta by Ang-II (100 n*M*, n = 9). Values are mean ± SE. * p < 0.05.

Co-application of the AT_1_ receptor antagonist losartan (10 n*M*) with Ang-II (100 n*M*) significantly and reversibly attenuated the Ang-II (100 n*M*)-evoked vasoconstriction of vasa recta at pericyte sites (15.3 ± 1.9%) by ∼30% to 10.9 ± 2.2% (p < 0.05, n = 8 slices, n = 3 animals; fig. 7). Higher concentrations of losartan (100 n*M*) irreversibly abolished the Ang-II-evoked vasoconstriction of vasa recta at pericyte sites (n = 9 slices, n = 3 animals; fig. 7). Superfusion of live kidney slices with the AT_1_ receptor antagonist losartan alone, at both 10 and 100 n*M* had no significant effect on vasa recta diameter at pericyte sites (fig. 7). No significant change in vasa recta diameter was observed at non-pericyte sites during these experiments (data not shown).

### Attenuating Endogenous NO and PGE_*2*_ Release in Kidney Slices

**Fig. 8:**
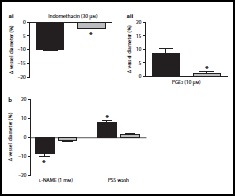
Attenuating endogenous release of NO and PGE_2_ in kidney slices. Superfusion of live kidney slices with the COX inhibitor indomethacin (30 µ*M*) evoked a significantly greater constriction of vasa recta at pericyte sites (**ai**, black bars) compared with non-pericyte sites (**ai**, grey bars, n = 13). Superfusion of PGE_2_ (10 µ*M*) evoked a significantly greater dilation of vasa recta at pericyte sites (**aii**, black bars) compared with non-pericyte sites (**aii**, grey bars, n = 5). Superfusion of the NOS inhibitor *L*-NAME (1 m*M*) caused a significantly greater constriction at pericyte sites (**b**, black bars) than at non-pericyte sites (**b**, grey bars, n = 7). Washout of *L*-NAME with PSS caused a significantly greater dilation of vasa recta at pericyte sites (**b**, black bars) than at non-pericyte sites (**b**, grey bars, n = 5). Values are mean ± SE. * p < 0.05.

Indomethacin and *L*-NAME were applied to kidney slices to attenuate the endogenous release of prostaglandins and NO, respectively. Application of the non-selective cyclooxygenase inhibitor indomethacin (30 µ*M*) evoked a significantly greater constriction of vasa recta at pericyte sites (9.9 ± 0.5%) than at non-pericyte sites (2.1 ± 0.3%, n = 13 slices, n = 5 animals, p < 0.05; fig. 8ai) that was consistent with attenuation of production of vasodilator prostaglandins such as PGE_2_. This effect was reversible following removal of indomethacin. Application of PGE_2_ to kidney slices evoked dilation of vasa recta that was significantly greater at pericyte sites than at non-pericyte sites (8.6 ± 1.6 and 1.0 ± 0.8% respectively, n = 5 slices, n = 4 animals; p < 0.01; fig. 8aii).

Application of 1 m*ML*-NAME (to inhibit nitric oxide synthase and NO production) evoked a significantly greater constriction of vasa recta at pericyte sites than at non-pericyte sites (8.6±.1.1 vs. 1.9 ± 0.3%, p < 0.05, n = 7 slices, n = 4 animals; fig. 8b) that reversed after removal of *L*-NAME. In some experiments following the removal of *L*-NAME, we observed a dilation of vasa recta at pericyte sites (8.0 ± 1.0%), which was significantly greater than at non-pericyte sites (1.9 ± 0.5%, n = 5 slices, n = 3 animals, p < 0.05; fig. 8b). This may be due to a surge in NO production following removal of *L*-NAME.

### Stimulating Endogenous Release of NA and ATP – Demonstrating Neurovascular and Tubulovascular Crosstalk

**Fig. 9:**
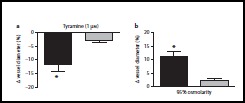
Stimulating the release of endogenous vasoactive mediators. Superfusion of kidney slices with tyramine (1 µ*M*) evoked a significantly greater constriction at pericyte sites (**a**, black bars) compared with non-pericyte sites (**a**, grey bars, n = 7). Reducing the osmolarity of the PSS by 5% caused a significantly greater dilation at pericyte sites (**b**, black bars) compared with non-pericyte sites (**b**, grey bars, n = 5). Values are mean ± SE. * p < 0.05.

Superfusion of live kidney slices with 1 µ*M* tyramine (to stimulate NE and ATP production and release from sympathetic nerve terminals) caused a significantly greater vasoconstriction of vasa recta at pericyte sites (11.9 ± 2.5%) compared with non-pericyte sites (3.0 ± 0.6%, n = 7 slices, n = 4 animals; p < 0.05; fig. 9a). This vasoconstriction was however more moderate than that observed following bath application of 10 n*M* NE (∼21%).

Tubular epithelial cells and vascular endothelial cells are both known to release vasoactive ATP under conditions of ‘stress’, such as hypotonicity. Reducing the osmolarity of the PSS by 5% in the current study caused a significant increase in vasa recta diameter at pericyte sites (11.4 ± 1.7) compared with non-pericyte sites (2.2 ± 0.9, n = 5 slices, n = 4 animals; p < 0.05; fig. 9b).

## Discussion

The purpose of this study was to develop and explore the potential for a *live* kidney slice model to investigate vasa recta properties and regulation in situ. Although the vasa recta are not perfused in this preparation, it has the advantage of being intact tissue with both vasa recta and adjacent tubules, and preserved intrarenal innervation; as such is potentially useful for examining the role of vasa recta pericytes in the control of vasa recta capillary diameter, and the effect of exogenously applied and endogenously released vasoactive compounds. Moreover, in the absence of an in vivo method to study vasa recta function in situ, we have shown that the kidney slice model confirms many of the observations already made in isolated vasa recta in vitro, and that the slice model may complement and extend current in vitro techniques.

### Tissue Viability

Kidney slices are viable and have an 80% ratio of live to dead cells using Hoechst and PI. This is similar to the ratio reported previously for healthy rat kidneys [23], and a value that was also obtained in the established rat brain slice preparation [7]. Furthermore, the uptake of calcein into both vascular and tubular cells in the renal medulla is not dissimilar to that in the renal cortex of transverse cut rat kidney slices shown by Hall et al. [24]. These findings indicate that kidney slice tissue is viable for physiological experiments.

### Characteristics of in situ Vasa Recta and Their Pericytes

Using anti-NG2 antibody to identify pericytes [25] in the renal medulla and to investigate the characteristics of in situ pericytes on vasa recta capillaries (identified with IB_4_) in live kidney slices, we found a significantly higher density of pericytes in the outer medulla compared with the inner medulla – similar to previous reports in sections of tissue dissected from the inner and outer medulla of rat kidneys [26]. We also found a wide range of 1° process lengths (2–18 µm) extending from the cell body and running in parallel along, and wrapping around, the vasa recta. Given that the average distance between pericyte cell bodies is ∼16 µm, and the average process length is ∼8 µm, there are unlikely to be many regions along the vasa recta that are not covered with pericyte processes, which probably account for the small changes in vasa recta diameter (<7%) at non-pericyte sites when Ang-II and NE were applied (fig. 4c and 5 ai, respectively); endothelial cells are unlikely to contract and cause changes in vessel diameter.

Interestingly, the density of pericytes on vasa recta is greater than that reported for many other tissues (for example in the mouse, CNS pericytes reside mainly at capillary branch points and have a distance of ∼50 µm between cell bodies [27]). The fluorescently conjugated IB_4_ used to label vasa recta was not able to distinguish between AVR and DVR in our kidney slices, and we cannot comment on AVR/DVR vessel diameter or pericyte density on DVR versus AVR.

In this study, sympathetic nerves were identified in close proximity to outer medullary pericytes. Since sympathetic nerves release NE, it is likely that these are an endogenous source of vasoactive NE in the medulla. When applied exogenously to kidney slices, 10 n*M* NE elicited vasoconstriction of vasa recta specifically at pericyte sites (see Results and fig. 5ai–iv). The application of tyramine also evoked pericyte-mediated vasoconstriction of in situ vasa recta. Given the close proximity of sympathetic nerves to vasa recta pericytes, we propose that tyramine stimulates local release of NE and ATP from sympathetic varicosities, and that this acts at apposite pericytes to cause pericyte contraction and constriction of vasa recta at that site. The constriction observed in tyramine experiments was however more moderate when compared with the pericyte-mediated constriction of vasa recta evoked by bath application of 10 nM NE. This is not perhaps surprising given that local concentration of NE has previously been determined for both the iris and vas deferens and is in the picomolar range, and thus significantly <10 n*M*[28].

Vasa recta capillaries in our study measured 7–10 µm in diameter, which is smaller than others have reported. Holliger et al. [29] reported a vessel diameter for both AVR (20 µm) and DVR (15 µm), when investigating vasa recta in the exposed papilla of young rats, and more recently vasa recta used in an in vitro microperfusion study were reported to have diameters of 13–15 µm [9,30]. The mean resting vessel diameter of non-perfused isolated DVR (pericyte vs. non-pericyte sites not defined in this early publication) is reported to be ∼11.5 ± 1 µm, which increases to 15.5 µm when DVR are perfused with a perfusion pressure up to 75 mm Hg. When perfused at a more physiological value of 25 mm Hg, mean isolated perfused DVR diameter is ∼14.5 µm [30], which is much closer to the values we determined for the outer vessel diameter (12.6 ± 0.5 µm) in our preparation. However, the measured diameters of microvessels in the present study are for the inner diameter and not the outer (which is usually the value given for isolated perfused DVR). The mean diameter of vasa recta at pericyte and non-pericyte sites was calculated to be ∼7.5 and ∼9.5 µm, respectively. Given that endothelial cells are generally considered to be 1–2 µm thick, this could increase vessel diameter to 9.5–11.5 and 11.5–13.5 µm at pericyte and non-pericyte sites, respectively.

### Functional Experiments on Pericytes

Agonist-evoked constriction of individual vasa recta in situ by pericytes can be measured in real time (see online suppl. video 1 as an example). Moreover, we found that endogenous signalling mechanisms are functioning in this kidney slice model, and that endogenous agents (NO and PGE_2_, NE and ATP) are acting via contractile pericytes to regulate vasa recta diameter, and changes in vessel diameter were significantly greater at pericyte sites compared with non-pericyte sites in all our experiments. Agonist-evoked changes in vasa recta diameter were observed at a small proportion of pericyte sites in each vessel, and in total occurred in approximately 51% of all vessels examined. The lack of response of some pericytes here is similar to findings on isolated and in situ retinal vessels. A P2X7 agonist contracted 37% of isolated retinal pericytes and capillary constriction occurred in only 12% of the total [31]; a muscarininc agonist contracted just 10% of retinal pericytes [32]. In whole mount retina, UTP and ATP constricted 30 and 25% of total pericytes, respectively [7]. In cerebellar slices, NA constricted 50% of pericytes, and only 20% of pericytes responded to glutamate [7]. The ranging success rate (35–83%) we observed in kidney tissue is in line with, or greater than, that observed in CNS experiments [7,31,32] (which is presumably in part due to the relative potency of the respective agonists used and whether they are acting at direct receptors, or stimulating endogenous release of other vasoactive compounds), and this finding correlates well with our observation that pericyte density is greater along vasa recta capillaries than along CNS capillaries. We suggest that if all pericytes were to constrict uniformly as a syncytium, the resulting vasoconstriction and reduction in medullary blood flow would be quite profound and detrimental to the already borderline hypoxic tissue. We believe that pericytes act to regulate vasa recta diameter in a finite way, offering regional redistribution of blood flow and fine-tuning of blood flow, as opposed to causing complete cessation of blood flow. The fact that not all pericytes appear to constrict, both in our preparation and in other tissue beds investigated to date, does seem to support this concept.

Agonist-evoked changes in vessel diameter were reversible and vessels returned to ∼97% of their original resting diameter following removal of all agonists, except for ET-1, where vasa recta diameter only returned to 76.5 ± 5.9%. Our findings agree with previous reports that Ang-II, NE and ET-1 significantly reduce blood flow in the medulla [1]. In our experiments we considered an agent more likely to reduce medullary blood flow when vasa recta diameter (normally ∼10 µm) is decreased enough to impede the passage of red blood cells (RBCs; diameter of 7–8 µm), for example Ang-II (100 n*M*) caused a 29% decrease in vasa recta diameter, which could significantly impede the flow of RBCs.

Ang-II-evoked vasoconstriction was unusual in that vessels started to dilate (toward their resting diameter) before the removal of Ang-II. This indicates possible desensitization of AT_1_ receptors, perhaps due to release of vasodilatory NO. AT_1_ receptor-stimulated release of NO has been reported in mouse afferent arterioles [33]; this effect has also been observed in isolated rat DVR in which chronic infusion of Ang-II enhanced basal production of NO [34]. Since Ang-II is endogenous to the kidney, it is likely that local vasodilatory mechanisms exist to counteract excessive Ang-II-induced vasoconstriction. Indeed, we have shown that (unperfused) vasa recta in kidney slices are capable of producing NO. Application of *L*-NAME, an inhibitor of NOS and NO production, caused vessels to constrict, suggesting that NO is released tonically in kidney slices and that it may determine resting vessel diameter. In experiments where kidney slices were exposed to Ang-II and the NO donor SNAP, SNAP attenuated the Ang-II-evoked constriction of vasa recta at pericyte sites when the Ang-II concentration was low (10 n*M*), but not at a higher concentration (100 n*M*).

Another possible trigger for local NO release in the slice preparation are RBCs present in the lumen of ∼50% of all vasa recta exposed to Ang-II (fig. 4ai–iii). Since RBCs are known to release ATP in response to deformation caused by vessel constriction [35], and that ATP can then lead to release of NO from the vascular endothelium [36], it is possible that pericyte-mediated constriction of vasa recta containing RBCs could result in ATP-mediated release of vasodilatory NO. Of the vasa recta containing RBCs, 87.5% recovered prior to washout of Ang-II compared with vasa recta without RBCs, in which only 50% recovered prior to Ang-II washout. The AT_1_ receptor antagonist losartan, at 100 n*M*, fully and irreversibly blocked Ang-II-evoked vasoconstriction of vasa recta at pericytes, whereas 10 n*M* losartan only partially blocked this effect. Thus, our findings are consistent with data collected in similar experiments on isolated DVR [37]. Inhibition of Ang-II-evoked constriction by losartan confirms AT_1_ receptors are present on the surface of vasa recta pericytes and probably mediate the Ang-II-evoked constriction of vasa recta by contracting pericytes.

In addition to attenuating endogenous production of NO, we have also shown that application of indomethacin caused vasa recta to constrict (see Results and fig. 8ai). Indomethacin is a selective COX-1 inhibitor and inhibits the synthesis of vasodilatory prostaglandins, including PGE_2._ We have demonstrated here that PGE_2_ evoked vasodilation of vasa recta at pericyte sites, this in combination with the indomethacin-evoked vasoconstriction suggests that the pericyte-mediated constriction observed in the presence of indomethacin is in part due to inhibition of vasodilatory PGE_2_.

In order to address the question of whether the live kidney slice model is suitable for investigating tubulovascular crosstalk, we sought to initiate ATP release from tubular epithelial cells by applying a hypotonic PSS solution to live kidney slices. Cultured tubular epithelial cells have previously been shown to release ATP under conditions of stress, such as pressure [38], and hypotonicity [39]. In the current study, we observed pericyte-mediated vasodilation in response to application of a hypotonic PSS indicative of ATP-mediated vasodilation. In cerebral vascular beds, ATP has been shown to cause both vasoconstriction and vasodilation [40,41]. Moreover, in the live kidney slice model, we have previously demonstrated that high concentrations of ATP (100 µ*M*) caused pericyte-mediated vasoconstriction [20]. In experiments where slices were exposed to inhibitors of the ATP-gated P2 receptors (suramin), we observed constriction of vasa recta at pericyte sites indicating low concentrations of ATP, such as that released locally, were tonically acting to dilate vasa recta [20]. Data presented here show that endogenous ATP release does indeed act to regulate vasa recta diameter; however, since we cannot demonstrate that the source of ATP release is solely tubular in its origin, findings presented here are perhaps the first step towards investigating tubulovascular crosstalk mechanisms in situ.

### Ang-II-Evoked Changes in Renal Blood Flow: in situ vs. in vitro vs. in vivo

To explore the value of this model as an in situ technique to investigate how medullary blood flow may be controlled locally, we compared changes in flow evoked by Ang-II in our in situ slice preparation with previously published in vitro isolated DVR experiments [9], and in vivo studies performed in our laboratory using intrarenal laser Doppler flowmetry (LDF) in rats [unpubl. data, see [42] for method]. Here we utilize Poiseuille's law to determine approximate changes in blood flow in response to Ang-II. Values presented are an approximation and determined by extrapolating data from measured changes in vessel diameter, therefore values are a simplification of the likely in vivo blood flow dynamic. Comparing concentration-matched data (10 n*M* Ang-II), we calculated an approximate 55% decrease in vasa recta blood flow in our slice experiments, which is less than that calculated for corresponding in vitro isolated DVR experiments (92%). Interestingly, in in vivo LDF experiments following the systemic infusion of Ang-II (3 µg/min/kg; which equates to 250 µl of ∼20 µ*M* Ang-II over 5 min; the maximum concentration that can be safely infused [unpubl. finding]), we observed only a 20.2 ± 5.4% (n = 9) change in medullary blood flow [unpubl. data]. This in vivo value is in keeping with other in vivo experiments reported in rats in which a 28% change in medullary blood flow was measured by LDF following Ang-II infusion [43]. Responses calculated for both the in situ slice model and in vitro DVR model are higher than the measured in vivo response, which is not surprising given the complex physiological regulation of blood in vivo; however, it is interesting that the value obtained using the slice preparation is intermediate between the in vitro and in vivo values, perhaps reflecting its relatively intact nature, though still lacking perfusion, as well as the normal osmotic and oxygen gradients present in vivo.

## Conclusion

Many of the limitations of the kidney slice model have already been alluded to. Ideally, an improved model would include vasa recta luminal perfusion, as well as simulation of the osmotic and oxygen gradients found in vivo, which are both technically challenging. However, despite these limitations, the current model may still have a useful place alongside the techniques currently available to study medullary blood flow, and may even be complementary.

We have shown that the kidney slice model is not a technically demanding preparation to set up, and that it can be used to demonstrate pharmacological effects on vasa recta pericytes, which are likely to have physiological relevance, and are not qualitatively dissimilar to what has been reported in vitro. However, what we see as the potential advantage of this technique is that is can be used to explore the structure-function relationship between the vasa recta and adjacent renal tubules, and the role of local innervation, not only in normal healthy renal tissue, but also in slices taken from models of acute kidney injury to investigate its pathophysiology.

## Figures and Tables

**Table 1:** Characterization of pericytes in the renal medulla

	Inner medulla	Outer medulla	Combined
Pericyte density (per 100 μm^2^)	9.1 ± 0.5*	12.4 ± 1.2	10.8 ± 0.7
Pericyte cell-body length, μm	8.9 ± 0.2	9.1 ± 0.2	9.0 ± 0.2
Distance between pericytes, μm	16.0 ± 1.2	15.1 ± 1.1	15.6 ± 1.8
1° process length, μm			
Along vessel	7.9 ± 0.5	8.9 ± 0.6	8.3 ± 0.4
Around vessel	0.3 ± 0.6	8.6 ± 0.5	9.0 ± 0.4
Combined	8.6 ± 0.4	8.7 ± 1.1	8.7 ± 0.3

Pericyte density, cell-body length, distance between pericytes, and 1° process lengths of the inner and outer medulla were calculated from confocal images of NG2-stained kidney slices (n = 3 slices per animal, n = 3 animals). Pericyte density was calculated from 100-µm^2^ regions in the inner and outer medulla. Values are mean ± SE.

* p < 0.05 inner vs. outer medulla.
